# Asthma

**Published:** 1856-12

**Authors:** 


*Asthma.*—Prof. Forget, of Strasbourg, in a memoir on nervous affections, and particularly asthma, arrives at the following conclusions:

1. Asthma is a special nervous affection, having a distinct existence like the other neuroses, even when it is developed consecutively to other affections, such as catarrh and emphysema.

2. Cases of primitive asthma, essential as they are termed, are, if not doubtful, sufficiently rare.

3. Almost always as soon as asthma is developed, it is connected with catarrh, sometimes with emphysema, and often with both, without these three affections necessarily being conjoined or resulting from the same cause.

4. In the great majority of cases catarrh and sometimes emphysema, and often both, precede for a long time the development of asthma so as to render it indubitable that the latter is consecutive.

5. Asthma is much rarer than catarrh and emphysema, which would not be true if these three affections were correlative.

6. Asthma, when once developed, may manifest itself rarely, at long intervals, or may disappear entirely, catarrh and emphysema persisting, a fact proving the specialty of asthma, but not its entire independence of the other affections.

7. The peculiar relations which asthma sustains to catarrh and emphysema may sometimes be explained by an analysis of clinical facts, and especially by the greater or less extent of the bronchitis.

8. The intermittency of asthma, the organic lesions being persistent, the most striking of its peculiarities, does not pertain exclusively to this affection, but also to all the neuroses.

9. The therapeutical deductions from these premises are, that asthma is not to be combatted always by the same measures; that the application of specifics is often irrational and illusory; that each of the constituent affections in particular cases, claims its share of curative means according to its predominance or its influence on the other affections, and that in controlling certain united affections we may often succeed in destroying the nervous affection.

10. In conclusion, by a kind of Providential provision, there is a remedy suited to each of these affections. This remedy is opium, which is equally appropriate to the catarrh, emphysema and the nervous affection.—*Gazette Hebdomadaire*, from *Annuaire des Sciences Médicales.* 1856.

